# Proteomics of aging: biomarkers for physiological systems and diseases

**DOI:** 10.3389/fragi.2026.1895104

**Published:** 2026-08-14

**Authors:** Ying Luo, Yu-Lin Xiao, Jie-Hua Chen, Hongjue Wang, Shuhong Luo, Hua Dong, Ruo-Pan Huang

**Affiliations:** 1 Department of Biomedical Engineering, School of Materials Science and Engineering, South China University of Technology, Guangzhou, China; 2 RayBiotech Co., Ltd., Guangzhou, China; 3 South China Biochip Research Center, Guangzhou, China; 4 Department of Urology, The Third Affiliated Hospital of Sun Yat-sen University, Guangzhou, China; 5 China-UK Joint Laboratory on Health and Ageing Research, Guangzhou, China; 6 Infinitus (China) Company Ltd., Guangzhou, China; 7 RayBiotech Inc., Peachtree Corners, GA, United States; 8 National Engineering Research Center for Tissue Restoration and Reconstruction (NERC-TRR), Guangzhou, China; 9 Affiliated Cancer Hospital and Institute of Guangzhou Medical University, Guangzhou, China

**Keywords:** aging, biological aging clock, biomarkers, inflammaging, proteomics

## Abstract

Aging is a progressive, multisystem process characterized by declining physiological resilience and increased susceptibility to chronic diseases. Recent advances in high-throughput proteomics have enabled comprehensive mapping of age-related changes across circulating proteins, revealing dynamic and non-linear trajectories that reflect biological rather than chronological aging. This review synthesizes current evidence on proteomic biomarkers across major physiological systems, including the immune, metabolic/endocrine, cardiovascular, musculoskeletal, and nervous systems, and highlights shared molecular signatures that underpin multisystem decline. Robust biomarkers such as IL-6, CRP, CXCL9/10, GDF15, IGF-1, VCAM-1, NT-proBNP, NfL, and GFAP consistently track inflammatory activation, mitochondrial and metabolic stress, extracellular matrix remodeling, and neuro-glial injury. Large population cohorts demonstrate that proteomic aging clocks, leveraging dozens to hundreds of circulating proteins, can predict frailty, multimorbidity, organ-specific biological age, and mortality with high accuracy. Emerging evidence suggests that a limited set of cross-system “protein aging modules”—including inflammatory cytokines, chemokines, complement proteins, and ECM-modifying enzymes—may serve as integrative readouts and potential regulators of aging biology. We discuss methodological advances, system-specific mechanisms, and translational applications of proteomic aging models. Together, these findings position proteomics as a powerful tool for quantifying biological age, identifying early disease risk, and guiding precision interventions to promote healthier aging.

## 1 Introduction

Aging is a pervasive and complex biological process that affects almost all organs and systems. It leads to decreased functional reserves, imbalances in homeostasis, and a significantly increased risk of various age-related diseases, including cardiovascular disease, metabolic syndrome, osteoporosis, and neurodegenerative diseases (Fabbri et al., 2015; Preston and Biddell, 2021; Guo et al., 2022; Tenchov et al., 2024). A substantial proportion of aging research has focused on genetic and transcriptomic alterations. However, changes at these levels do not fully reflect the actual abundance of proteins, their post-translational modification status, and their secretion and clearance in body fluids (Tanaka et al., 2020; Kliuchnikova et al., 2024). In contrast, proteomic approaches interrogate protein abundance and, depending on the platform, peptide sequences, PTMs, and proteoforms, thereby providing molecular readouts that are more proximal to functional phenotypes. As a result, proteomics has emerged as a key approach for quantifying biological age, identifying aging-related pathways, and discovering clinically relevant biomarkers (Ma et al., 2025).

Recent large-scale population studies have shown that the circulating proteome exhibits a highly specific and non-linear age trajectory throughout the lifespan (Lehallier et al., 2019; Argentieri et al., 2024; Shen et al., 2024). For example, Lehallier et al. measured approximately 2,925 plasma proteins in 4,263 individuals aged 18–95 years and found significant peaks in proteomic changes around ages 34, 60, and 78, corresponding to inflection points in the functions of different physiological systems. The same study derived a sex-independent 373-protein age model whose deviation from chronological age was associated with clinical and functional measures, supporting the view that proteomic age captures heterogeneity in health status rather than age alone. These findings collectively indicate that aging has quantifiable and modelable systemic characteristics at the proteomic level (Johnson et al., 2020; Salignon et al., 2023; Zhao et al., 2024).

The clinical relevance of these associations was further quantified in the InCHIANTI cohort. Among 997 participants aged 21–102 years profiled for 1,301 plasma proteins, Tanaka et al. identified 651 age-associated proteins and validated substantial directional concordance in independent cohorts (Tanaka et al., 2020). A previously derived 76-protein signature correlated strongly with chronological age (r = 0.87), while proteomic age acceleration predicted all-cause mortality over an 18-year follow-up independently of chronological age (hazard ratio per standard deviation, 1.29; 95% confidence interval, 1.11–1.50). Moreover, approximately one-third of age-associated proteins predicted mortality and 45.3% were associated with multimorbidity. These results provide effect-size context for the utility of proteomic aging signatures, but also illustrate a central specificity challenge: highly informative markers such as GDF15, NPPB, and inflammatory proteins can reflect concurrent cardiovascular, renal, metabolic, or malignant disease as well as aging itself (Tanaka et al., 2020).

Studies at the level of individual organs or systems have shown that many proteins related to immune responses, inflammation, extracellular matrix (ECM), and metabolic and endocrine regulation undergo consistent changes during aging (Tanaka et al., 2020; Santinha et al., 2024). For example, in immunosenescence and inflammatory senescence, cytokines and chemokines are highly enriched in age-related protein lists. In the cardiovascular and skeletal systems, ECM remodeling enzymes and structural proteins undergo remodeling. Additionally, in metabolic and endocrine senescence, stress factors such as GDF15 are among the most significantly altered plasma proteins (Coenen et al., 2023; Evans et al., 2024; Kamalian et al., 2025; Salminen, 2025). These recurring patterns suggest that multisystem aging may be organized around a relatively limited set of shared protein modules, rather than representing a simple aggregation of independent organ-specific changes.

This review aims to move beyond simply cataloging age-associated proteins and provide a system-level framework for understanding proteomic aging. First, we organize circulating proteomic changes across major physiological systems—including the immune, metabolic/endocrine, cardiovascular, musculoskeletal, and nervous systems—while highlighting both system-specific features and shared molecular patterns. Second, we integrate evidence across these systems to identify cross-system protein modules, such as inflammatory cytokines (e.g., IL-6), stress-responsive factors (e.g., GDF15), chemokines (e.g., CXCL9/10), and extracellular matrix–modifying enzymes, that may serve as common regulators or readouts of multisystem aging. Finally, we discuss methodological considerations, the biological interpretation of proteomic aging models, and the translational implications of high-throughput proteomics, positioning circulating proteins as integrative biomarkers linking molecular aging processes to functional decline across organ systems.

## 2 Immune system (immunosenescence and inflammatory senescence)

Inflammaging is a central feature of immune aging and a major contributor to age-related functional decline. The aging immune system undergoes two tightly interconnected processes: immunosenescence and chronic low-grade inflammation (Thomas et al., 2020; Goyani et al., 2024). Immunosenescence is characterized by reduced output of naive T cells due to thymic involution, narrowing of the TCR repertoire, accumulation of late-differentiated or exhausted memory/effector T cells, as well as B-cell dysfunction and increased autoantibody production. In parallel, inflammaging is marked by persistent low-level systemic inflammation, which weakens host defense and vaccine responsiveness while promoting tissue damage and age-related disease (Ferrucci and Fabbri, 2018; Ma et al., 2019; De Mol et al., 2021; Santoro et al., 2021; Yu et al., 2024).

Plasma and serum proteomics studies have identified numerous secreted proteins that serve as molecular signatures of immunosenescence and inflammaging. Among them, IL-6, CRP, CXCL9, and CXCL10 are among the most robust circulating biomarkers, consistently increasing with age and showing strong associations with frailty, multimorbidity, and all-cause mortality (De Oliveira Neto et al., 2021; Sayed et al., 2021; St Sauver et al., 2022). IL-6 is often regarded as a prototypical aging cytokine because it links chronic inflammatory activation to functional decline, whereas CXCL9 has emerged as a particularly informative marker of inflammatory aging, remaining relatively low in early adulthood but rising sharply later in life. In the iAge study, deep immune profiling of 1,001 individuals aged 8–96 years generated an inflammatory aging clock associated with multimorbidity, frailty, immunosenescence, and cardiovascular aging; CXCL9 was the strongest contributor and was linked experimentally to endothelial dysfunction (Sayed et al., 2021). Together, these proteins define a recurrent inflammatory module that extends beyond immune aging itself and recurs across multiple proteomic aging studies (Walker et al., 2022; Seo et al., 2024; Tylutka et al., 2024).

In addition to canonical inflammatory cytokines, several non-cytokine proteins also reflect immune aging. β2-microglobulin (B2M), a component of MHC class I molecules, accumulates with age and is associated with frailty and cognitive decline, supporting its role primarily as a biomarker of immune and systemic aging (Smith et al., 2015; Jheng et al., 2024; Ke et al., 2025). By contrast, GDF15, a stress-induced member of the TGF-β superfamily, may be interpreted more broadly as a candidate mediator linking mitochondrial dysfunction, altered immune regulation, and organismal stress responses (Kochlik et al., 2023; Wang et al., 2024a).

Large-scale aging proteomics further supports the centrality of immune and inflammatory pathways. In human plasma proteome studies, immune-related proteins—including cytokines, complement components, and acute-phase reactants—represent a major fraction of age-associated proteins, and their trajectories are distinctly non-linear across the lifespan (Lehallier et al., 2019; Johnson et al., 2020). More recent proteomic aging clock studies similarly show enrichment of inflammatory and immune-response pathways among proteins that best predict biological age, reinforcing the idea that these molecules reflect shared aging biology rather than isolated immune alterations (Sayed et al., 2021).

At the mechanistic level, inflammaging arises from multiple upstream processes. Senescent cells and the senescence-associated secretory phenotype (SASP) constitute a major source of circulating inflammatory signals, releasing cytokines, chemokines, and proteases that propagate local damage into systemic inflammation (Ferrucci and Fabbri, 2018; Franceschi et al., 2018; López-Otín et al., 2023). Additional contributors include mitochondrial dysfunction and mtDNA leakage, chronic viral antigenic stimulation, and gut barrier deterioration with microbiome-associated inflammation (Franceschi et al., 2018). Within this context, the complement cascade represents a particularly important component of age-related immune remodeling.

Complement proteins such as C1q, C3, and C4 show sustained age-associated increases across multiple cohorts and are increasingly recognized as a cross-system hub rather than merely markers of immune activation (Naito et al., 2012; Franceschi et al., 2018; Scott-Hewitt et al., 2024). Their associations with vascular inflammation, muscle decline, and synaptic pruning suggest that complement activation may connect immunosenescence with functional deterioration across cardiovascular, musculoskeletal, and nervous systems (Naito et al., 2012; Stephan et al., 2012; Hong et al., 2016). Thus, inflammatory and complement-related proteins should be viewed not only as indicators of immune aging but also as biologically meaningful components of a shared circulating module that contributes to multisystem aging (Naito et al., 2012; Franceschi et al., 2018).

## 3 Cross-system metabolic and endocrine remodeling

As individuals age, systemic energy metabolism undergoes progressive remodeling, including visceral fat accumulation, adipose inflammation, increased insulin resistance, reduced glucose utilization, declining basal metabolic rate, impaired mitochondrial oxidation, reduced ATP production, and dysregulated lipid metabolism with increased lipotoxicity (Amorim et al., 2022; Ou et al., 2022; Palmer and Jensen, 2022). Together, these changes define metabolic aging. At its core, metabolic aging reflects a mismatch between energy production and energy demand, with consequences that extend beyond classical metabolic tissues through pathways involving insulin signaling, adipokines, and muscle-derived metabolic factors (Mancuso and Bouchard, 2019; Amorim et al., 2022).

Several major hormonal axes also change substantially with age. GH and IGF-1 decline after middle age, a phenomenon often referred to as somatopause. Reduced IGF-1 is associated with frailty, sarcopenia, cognitive decline, and increased mortality risk, consistent with reduced anabolic capacity and impaired tissue regeneration (Junnila et al., 2013; Milman et al., 2016). Aging of the gonadal axis likewise contributes to altered fat distribution, bone loss, reduced muscle mass, and dysregulated inflammation (Xia et al., 2017; Bartke, 2019; Cheng et al., 2024). In addition, elevated TSH, reduced T3, and age-related declines in DHEA and DHEA-S further reflect endocrine remodeling during aging and contribute to changes in body composition and substrate utilization (Papadopoulou-Marketou et al., 2000; Franceschi et al., 2019; Taylor et al., 2023). These observations indicate that metabolic aging is tightly linked to endocrine dysregulation rather than representing a purely metabolic phenotype.

At the molecular level, metabolic aging is closely associated with mitochondrial dysfunction, oxidative damage, and remodeling of nutrient-sensing pathways such as AMPK and mTOR (Li et al., 2024b; Somasundaram et al., 2024). Among circulating markers, IGF-1 remains one of the most established biomarkers of anabolic aging, with lower levels consistently linked to reduced muscle and bone function (Chu et al., 2025). Adipokines also provide important readouts of metabolic aging. Adiponectin often increases in older adults despite worsening metabolic status, giving rise to the so-called adiponectin paradox, whereas leptin may decline after peaking in midlife (Schautz et al., 2012; Walowski et al., 2023). These proteins are best interpreted as biomarkers of altered energy balance, body composition, and endocrine-metabolic adaptation (Schautz et al., 2012).

Among stress-responsive proteins, GDF15 has emerged as one of the most robust circulating markers of aging (Jena et al., 2023). Induced by mitochondrial dysfunction, nutritional stress, and inflammatory signaling, GDF15 shows a strong age-dependent increase and is consistently identified in large-scale proteomic aging studies (Torrens-Mas et al., 2025). Its associations with weight loss, anorexia, insulin resistance, and impaired cardiac function suggest that it captures a broader cross-system stress program linking metabolic imbalance, inflammation, and tissue remodeling (Ouyang et al., 2020; Wang et al., 2023; Li et al., 2024a). In this regard, GDF15 may be more informative than a conventional metabolic biomarker and may also represent a candidate mediator of organismal stress responses (Ouyang et al., 2020; Li et al., 2024a).

High-throughput proteomics has also identified other endocrine-related factors with potential mechanistic relevance (Ma et al., 2025). In particular, Mendelian randomization analyses suggest that IGFBP4, an inhibitor of IGF-1 signaling, may represent a more causally implicated aging-related protein than markers that function primarily as correlates or readouts (Ma et al., 2025). Furin, whose activity increases with age, may further contribute to metabolic imbalance by aberrantly activating cytokines and growth factors (Wichaiyo et al., 2024; Ma et al., 2025). In addition, elevated SHBG reflects reduced sex hormone bioavailability, whereas FGF21, a key mitochondrial stress hormone, increases in older adults and may reflect a compensatory response to impaired metabolic homeostasis (Aribas et al., 2021; Burtscher et al., 2023).

**FIGURE 1:**
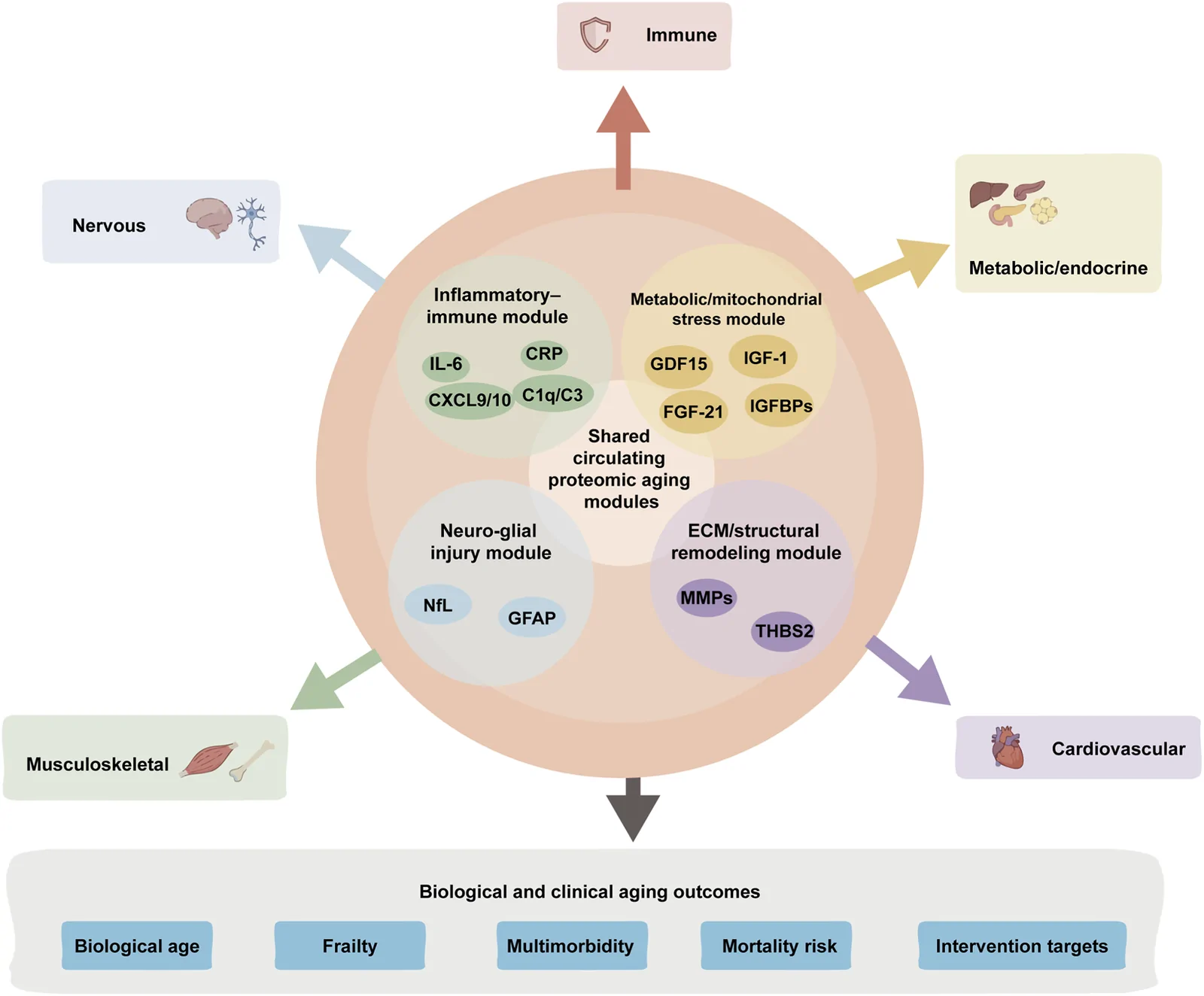
Conceptual framework of shared circulating proteomic aging modules linking multisystem decline to biological and clinical aging outcomes. Circulating proteomic aging can be conceptualized as a limited set of recurrent cross-system modules rather than isolated organ-specific signatures. Four major shared modules are highlighted here: an inflammatory–immune module (represented by IL-6, CRP, CXCL9/10, and C1q/C3), a metabolic/mitochondrial stress module (GDF15, IGF-1, IGFBPs, and FGF21), an extracellular matrix (ECM)/structural remodeling module (MMPs and THBS2), and a neuro-glial injury module (NfL and GFAP). These modules interact with multiple physiological systems—including the immune, metabolic/endocrine, cardiovascular, musculoskeletal, and nervous systems—and collectively contribute to biological age acceleration, frailty, multimorbidity, mortality risk, and the identification of cross-system intervention targets.

Overall, metabolic and endocrine aging is shaped by a multilayered circulating protein network composed of hormones, binding proteins, adipokines, stress-response factors, and metabolic regulators (Kliuchnikova et al., 2024; Oh et al., 2025). Rather than reflecting isolated endocrine decline, these proteins define a shared metabolic-stress module that links energetic imbalance, inflammatory activation, musculoskeletal decline, and cardiovascular dysfunction (Oh et al., 2023). This conceptual framework is illustrated in Figure 1.

## 4 Cardiovascular aging as an integrative outcome system

Cardiovascular disease remains a leading cause of morbidity and mortality in older adults. Even in the absence of overt pathology, the cardiovascular system undergoes characteristic age-related changes, including arterial stiffness, endothelial dysfunction, and cardiac remodeling, which together define the core phenotype of cardiovascular aging (Li et al., 2023a; Müller and Di Benedetto, 2024; Tana et al., 2025). Increased elastic fiber fragmentation and collagen deposition reduce large-artery compliance, while endothelial nitric oxide bioavailability declines and shifts toward a pro-inflammatory and pro-thrombotic state (Li et al., 2023a; Müller and Di Benedetto, 2024; Adhikari et al., 2025). In parallel, the aging heart exhibits left ventricular hypertrophy, fibrosis, and impaired functional reserve, thereby increasing susceptibility to hypertension, atherosclerosis, and heart failure with preserved ejection fraction (HFpEF) (Kim and Jo, 2024; Müller and Di Benedetto, 2024).

At the circulating proteome level, cardiovascular aging is reflected by several recurrent classes of proteins. VCAM-1 is among the most representative markers of endothelial activation, and its soluble form increases with age, supporting its use as a readout of vascular inflammation and endothelial dysfunction (Yousef et al., 2019; Pickett et al., 2023). This vascular inflammatory signal may also contribute to cerebrovascular dysfunction and cognitive decline, linking cardiovascular aging to brain aging (Tchalla et al., 2017; Yousef et al., 2019). By contrast, NT-proBNP and high-sensitivity cardiac troponins primarily reflect chronic myocardial wall stress and subclinical cardiac injury. Their age-dependent elevation, even in individuals without overt heart disease, suggests that they function predominantly as readouts of cardiovascular aging burden rather than established primary drivers of the aging process (Averina et al., 2022; Myhre et al., 2024; Heinrich et al., 2025).

Large-scale plasma proteomic studies further suggest that cardiovascular aging should be interpreted within a broader cross-organ context. Using 4,979 plasma proteins measured in 5,676 adults from five independent cohorts, Oh et al. developed aging models for 11 organs and found that nearly 20% of individuals exhibited strongly accelerated aging in a single organ, whereas only 1.7% showed multi-organ acceleration (Oh et al., 2023). Accelerated aging across individual organs was associated with higher all-cause mortality risk, while a one-standard-deviation increase in heart age gap was associated with an approximately 2.4-fold higher risk of incident heart failure over 15 years of follow-up (HR = 2.37). These organ-enriched signatures may provide greater anatomical resolution than a global proteomic age score, although they should not be interpreted as direct measurements of tissue-specific aging because organ assignment depends partly on reference transcriptomic data and the presumed tissue origins of circulating proteins. At the translational level, high-dimensional plasma proteomic models have also shown potential to improve cardiovascular risk stratification, including in high-risk populations such as individuals with chronic kidney disease (Deo et al., 2023; Royer et al., 2024; Qu et al., 2025).

Inflammatory and ECM-related proteins are particularly important in this context. IL-6 and CRP, discussed above as components of the shared inflammatory aging module, also contribute to cardiovascular risk by promoting endothelial dysfunction, coagulation-related responses, and acute-phase signaling (Kang and Kishimoto, 2021). THBS2, a matricellular glycoprotein involved in extracellular matrix remodeling and fibrosis, increases in the setting of cardiovascular burden and predicts adverse outcomes, including cardiovascular death and heart failure (Hanatani et al., 2014; Kimura et al., 2016). MMP-9, an ECM-degrading enzyme that is upregulated in senescent and remodeled tissues, is linked to arterial stiffness, aneurysm formation, and maladaptive vascular remodeling, suggesting that it may function not only as a biomarker but also as a candidate mediator of cardiovascular aging (Wang et al., 2015; Shen et al., 2025). This distinction is important because it separates proteins that primarily indicate cardiovascular aging burden from those that may participate more directly in the remodeling process itself.

Overall, cardiovascular aging is characterized by a circulating proteomic pattern that integrates endothelial activation, chronic inflammatory input, ECM remodeling, and myocardial injury (Li et al., 2023a; Tana et al., 2025). These signatures suggest that cardiovascular aging reflects both organ-specific vascular and cardiac remodeling and broader inflammatory, metabolic, and structural aging processes (Oh et al., 2023; Royer et al., 2024).

## 5 Musculoskeletal aging and functional decline

The musculoskeletal system undergoes coordinated degenerative remodeling during aging, with sarcopenia and osteoporosis representing its two principal clinical phenotypes (Miljkovic et al., 2015; Gielen et al., 2023). Both are major contributors to frailty, falls, and fracture risk in older adults (Greco et al., 2019; Gielen et al., 2023). In skeletal muscle, aging is characterized by preferential type II fiber atrophy, increased fat infiltration, impaired satellite-cell regenerative capacity, and neuromuscular junction degeneration, ultimately driving progressive declines in muscle mass and strength (Jang and Van Remmen, 2011; Khosa et al., 2019; Gielen et al., 2023).

Proteomic studies provide a more detailed molecular view of muscle aging. Circulating proteins involved in antioxidant defense and tissue maintenance, such as PON3, and potentially CD9, are reduced in older individuals with sarcopenia, consistent with impaired oxidative resilience and regenerative capacity (Lin et al., 2024b; Yin et al., 2025). At the same time, inflammatory myokines and cytokines, including IL-6 and, in some studies, IL-8, increase in aging and sarcopenia, reflecting a shift toward chronic catabolic and inflammatory signaling along the muscle–systemic axis (Wang et al., 2017; Ding et al., 2024; Cui et al., 2025). C-terminal agrin fragments (CAFs), which are released during neuromuscular junction degeneration, have emerged as candidate circulating biomarkers of NMJ instability and neuromuscular aging, although their associations with muscle mass and functional decline remain under active investigation (Scherbakov et al., 2016; Pratt et al., 2021; Fatima et al., 2025). Overall, these findings suggest that proteomic changes in aging muscle reflect not only local atrophy but also broader alterations in neuromuscular integrity, oxidative defense, and systemic catabolic signaling (Scherbakov et al., 2016; Wang et al., 2017; Moreira-Pais et al., 2022).

Age-related skeletal decline likewise reflects a shift toward bone resorption over bone formation. Postmenopausal women undergo accelerated bone loss due to estrogen deficiency, whereas men generally show a slower decline in bone mass (Khosla and Pacifici, 2013). Molecular studies of aging bone indicate substantial remodeling of the extracellular matrix, including alterations in collagen, proteoglycans, and non-collagenous structural proteins, consistent with reduced matrix integrity and increased fragility (Sroga and Vashishth, 2012; Ravazzano et al., 2024). In parallel, osteoclast-associated factors such as Cathepsin K, CTX-I, and RANKL are elevated in age-related bone resorption states, whereas markers of bone formation decline (Bonnet et al., 2020; Moon, 2024). These proteins are therefore best interpreted as circulating readouts of imbalance in bone turnover and matrix remodeling during skeletal aging (Moon, 2024).

Importantly, muscle and bone do not age independently. Increasing evidence supports a coupled aging process within the muscle–bone unit, in which shared inflammatory, metabolic, and endocrine signals contribute to both sarcopenia and osteoporosis (Novotny et al., 2015; Lu et al., 2021). For example, IGFBP-2 has been linked to low bone mineral density and has also been implicated in adverse musculoskeletal phenotypes, while IL-6 and TNF-α can promote both muscle atrophy and osteoclast activation (Amin et al., 2004; Khosla and Pacifici, 2013; Lu et al., 2021). Together, these findings support the view that musculoskeletal aging is best understood as a cross-system structural and catabolic module rather than as two isolated tissue-specific processes (Novotny et al., 2015; Lu et al., 2021). In this context, some proteins primarily function as biomarkers of tissue decline, whereas others, particularly inflammatory and resorption-associated factors, may also act as candidate mediators of coupled muscle–bone deterioration (Khosla and Pacifici, 2013; Lu et al., 2021).

Overall, musculoskeletal aging exhibits a characteristic circulating proteomic pattern marked by reduced structural and antioxidant support, increased inflammatory and catabolic signaling, neuromuscular junction instability, and a shift toward bone resorption and matrix disorganization (Lin et al., 2024b; Bhattoa et al., 2025). These proteins complement conventional indicators such as bone mineral density and bone turnover markers, providing a broader framework for understanding frailty-related decline across muscle and bone.

## 6 Neurological aging and neurodegeneration

Aging is a major contributor to the progressive decline of brain structure and function. Even in apparently healthy individuals, aging is accompanied by impaired synaptic plasticity, white matter degeneration, reduced integrity of myelinated axons, and gradual decline in processing speed, memory, and executive function (Navakkode and Kennedy, 2024; Groh and Simons, 2025). At the molecular level, the aging brain exhibits a characteristic neuroinflammatory state, including chronic low-grade microglial activation, increased complement-related activity, and a progressively more pro-inflammatory tissue environment (Li et al., 2023b; Scott-Hewitt et al., 2024; Müller et al., 2025). In parallel, the accumulation of misfolded proteins such as Aβ, tau, α-synuclein, and TDP-43 increases vulnerability to neurodegenerative disease (Pradeepkiran et al., 2025).

Among circulating and cerebrospinal fluid biomarkers, Aβ42 and tau remain central markers of Alzheimer’s disease pathology, although their changes are generally more pronounced in disease than in normal aging (McGrowder et al., 2021; Wang et al., 2024c; Pradeepkiran et al., 2025). In contrast, neurofilament light chain (NfL) has emerged as a more broadly applicable biomarker of neuroaging. NfL increases with age in both CSF and plasma and rises further in neurodegenerative disorders, where it tracks brain atrophy, cognitive decline, and disease progression (Götze et al., 2024; Hofmann et al., 2024; Zhang et al., 2025). Similarly, GFAP, and particularly CSF sTREM2, also increase with age and show further elevations in conditions such as AD, reflecting astroglial and microglial activation, respectively (Lin et al., 2024a; Wang et al., 2024d; Grande et al., 2025). With the development of ultrasensitive detection platforms, these axonal and glial injury markers can now be measured reliably in plasma, making peripheral monitoring of brain aging increasingly feasible (Nilsson et al., 2024; Grande et al., 2025).

Systematic proteomic studies of CSF, brain tissue, and plasma further suggest that neurological aging is shaped not only by neuronal injury markers but also by broader inflammatory and extracellular matrix remodeling pathways (Chmelova et al., 2023; Oh et al., 2025). Complement proteins, particularly C1q and C4-related components, are altered during normal aging and are further dysregulated in AD, supporting the idea that complement-mediated synaptic pruning contributes to synaptic loss and cognitive decline (Liu et al., 2025; Nimmo et al., 2024; Tenner and Petrisko, 2025). Likewise, remodeling of the neural extracellular matrix is increasingly recognized as an important component of brain aging (Chmelova et al., 2023; Oh et al., 2025). Decreased levels of perineuronal net–related components such as BCAN and NCAN, together with increased proteases such as MMP-9, suggest progressive destabilization of the synaptic microenvironment in individuals with accelerated brain aging (Chmelova et al., 2023; Sanchez et al., 2024; Oh et al., 2025). In this context, complement- and ECM-related proteins may be interpreted not only as biomarkers, but also as candidate mediators of age-related neural network instability (Chmelova et al., 2023; Nimmo et al., 2024; Tenner and Petrisko, 2025).

Overall, neurological aging is characterized by a multidimensional proteomic landscape that includes classical amyloid and tau pathology, axonal and glial injury markers, and broader complement-, ECM-, and inflammation-related pathways (Ma et al., 2025; Oh et al., 2025). Together, these proteins provide accessible readouts of brain aging and neurodegenerative risk, while also linking neural vulnerability to systemic inflammatory and structural remodeling processes (Casanova et al., 2024; Oh et al., 2025).

## 7 Cross-system integration: shared circulating proteomic aging modules

Although age-associated proteins are often described within individual organ systems, evidence from large plasma proteomic cohorts increasingly suggests that proteomic aging is organized around recurrent circulating modules rather than isolated organ-specific signatures (Argentieri et al., 2024; Ma et al., 2025; Oh et al., 2025). A summary of representative cross-system circulating proteomic markers, their affected systems, biological interpretation, and translational readiness is provided in Table 1. These shared modules help explain why similar proteins and pathways repeatedly emerge across aging studies, organ-specific analyses, and proteomic aging clocks.

**TABLE 1:** Representative cross-system circulating proteomic markers of aging.

Protein/marker	Primary biological function	Major module	Affected systems	Main aging association	Main supporting evidence	Evidence-informed interpretation	Translational readiness	Representative references
IL-6	Pro-inflammatory cytokine; JAK/STAT signaling	Inflammatory–immune	Immune, cardiovascular, musculoskeletal, nervous	Inflammaging, frailty, multimorbidity, functional decline	Cohort association; inflammatory aging studies	Biomarker with potential mechanistic relevance	High assay maturity; limited aging specificity	Sayed et al. (2021), St Sauver et al., 2022, Ding et al. (2024)
CRP	Acute-phase reactant and innate immune opsonin	Inflammatory–immune	Immune, cardiovascular, metabolic	Systemic inflammation, frailty, cardiometabolic risk	Cohort association; clinical biomarker studies	Primarily a biomarker/readout	High clinical maturity; nonspecific for aging	Sayed et al. (2021), St Sauver et al., 2022
CXCL9/CXCL10	CXCR3 ligands that recruit activated T and NK cells	Inflammatory–immune	Immune, cardiovascular, musculoskeletal, nervous	Chemokine activation, inflammaging, immune remodeling	Cohort association; network analyses	Biomarker with potential mechanistic relevance	Research-stage/moderate translational interest	Sayed et al. (2021), Walker et al. (2022), Seo et al. (2024)
Complement proteins (C1q/C3/C4)	Opsonization, inflammatory amplification, and synaptic pruning	Inflammatory–immune/complement hub	Immune, cardiovascular, musculoskeletal, nervous	Chronic immune activation, vascular inflammation, synaptic pruning, muscle decline	Cohort association; mechanistic studies	Candidate mediator	Moderate translational readiness	Naito et al. (2012), Stephan et al. (2012), Hong et al. (2016), Scott-Hewitt et al. (2024)
GDF15	Integrated stress-response cytokine/mitokine	Metabolic–mitochondrial stress	Immune, metabolic, cardiovascular, musculoskeletal	Mitochondrial stress, frailty, weight loss, insulin resistance, mortality risk	Cohort association; identified in proteomic aging models; mechanistic studies	Biomarker with potential mechanistic relevance	Emerging translational relevance	Ouyang et al. (2020), Conte et al. (2022), Evans et al. (2024)
IGF-1	Anabolic growth, survival, and tissue maintenance	Anabolic/endocrine decline	Metabolic, musculoskeletal, nervous	Somatopause, frailty, muscle and bone loss, functional decline	Cohort association; endocrine biology studies	Primarily a biomarker/readout	High assay maturity	Junnila et al. (2013), Milman et al. (2016), Chu et al. (2025)
IGFBP4	Regulation of IGF bioavailability and signaling	Endocrine regulation	Metabolic, cardiovascular, musculoskeletal	Modulation of IGF signaling; aging association	Cohort association; Mendelian randomization	Candidate causal marker	Emerging translational relevance	Evans et al. (2024), Ma et al. (2025)
FGF21	Endocrine control of glucose and lipid metabolism	Metabolic–mitochondrial stress	Metabolic, cardiovascular, musculoskeletal	Metabolic impairment, mitochondrial stress, systemic adaptation	Cohort association	Primarily a biomarker/adaptive readout	Moderate translational readiness	Burtscher et al. (2023), Jena et al. (2023)
VCAM-1	Leukocyte adhesion and endothelial activation	Endothelial activation	Cardiovascular, nervous	Vascular aging, endothelial dysfunction, cognitive aging	Cohort association	Biomarker with potential mechanistic relevance	Moderate translational readiness	Yousef et al. (2019), Pickett et al. (2023)
NT-proBNP	Inactive cleavage fragment released in response to myocardial wall stress	Cardiac stress/injury	Cardiovascular	Cardiac wall stress, subclinical dysfunction, mortality risk	Cohort association; clinical biomarker studies	Primarily a biomarker/readout	High clinical maturity	Averina et al. (2022), Myhre et al. (2024), Heinrich et al. (2025)
MMP-9	Extracellular-matrix proteolysis and remodeling	ECM remodeling/proteolysis	Cardiovascular, musculoskeletal, nervous	Arterial stiffness, ECM turnover, synaptic instability	Cohort association; mechanistic studies	Candidate mediator	Moderate translational readiness	Wang et al. (2015), Chmelova et al. (2023), Shen et al. (2025)
THBS2	Matrix organization and cell–matrix signaling	ECM remodeling	Cardiovascular, musculoskeletal	Structural remodeling, cardiovascular burden, tissue fragility	Cohort association	Biomarker with potential mechanistic relevance	Moderate translational readiness	Hanatani et al. (2014), Kimura et al. (2016)
NfL (NEFL)	Axonal cytoskeletal protein released after injury	Neuro-glial injury	Nervous	Axonal injury, cognitive decline, neurodegeneration, brain aging	Cohort association; identified in proteomic aging models; brain aging studies	Primarily a biomarker/readout	Increasing clinical uptake	Götze et al. (2024), Nilsson et al. (2024), Oh et al. (2025)
GFAP	Astrocytic intermediate filament and glial-injury marker	Neuro-glial injury	Nervous	Astroglial activation, brain aging, neurodegeneration	Cohort association; identified in proteomic aging models; brain aging studies	Primarily a biomarker/readout	Moderate to high translational readiness	Nilsson et al. (2024), Grande et al. (2025), Oh et al. (2025)

One major module is the inflammatory–immune axis, represented by IL-6, CRP, CXCL9/10, and complement proteins. These factors capture chronic immune activation that extends beyond immunosenescence itself and links systemic inflammation to vascular dysfunction, musculoskeletal decline, and neuroinflammatory remodeling (Sayed et al., 2021; Oh et al., 2025). In this sense, inflammatory proteins are best viewed not only as markers of immune aging, but also as integrative signals of multisystem decline.

A second recurring module involves metabolic and mitochondrial stress. Proteins such as GDF15, FGF21, and components of the IGF axis reflect energetic imbalance, anabolic decline, and systemic stress adaptation rather than isolated endocrine changes (Conte et al., 2022; Li et al., 2024a; Ma et al., 2025). Their repeated appearance in proteomic aging studies suggests that metabolic aging is best understood as a cross-system stress-response program linking adiposity, insulin resistance, frailty, and cardiometabolic dysfunction (Argentieri et al., 2024; Ma et al., 2025).

A third important module involves the extracellular matrix (ECM) and structural remodeling. Age-predictive proteins related to ECM interaction and cell adhesion highlight the cumulative remodeling of tissue architecture across multiple organs, including arterial stiffening in the cardiovascular system, matrix fragility in bone and muscle, and synaptic or perivascular destabilization in the aging brain. Conceptually, this ECM module provides a structural counterpart to inflammatory signaling by capturing the progressive loss of tissue resilience across organ systems (Oh et al., 2023; Oh et al., 2025).

A fourth module reflects neuro-glial injury and the peripheral representation of brain aging. Proteins such as NfL and GFAP indicate that circulating proteomic aging also captures axonal injury, glial activation, and neural tissue vulnerability. Importantly, this module does not stand apart from the others, as complement activation, inflammatory signaling, and vascular dysfunction all interact with brain aging biology (Oh et al., 2025).

Taken together, these observations support a modular view of proteomic aging, in which circulating proteins do not simply report parallel deterioration in separate organs, but instead reveal a partially shared architecture of organismal aging. This perspective also helps explain the biological meaning of proteomic aging clocks: their predictive power likely arises from integrating recurrent processes such as inflammatory burden, metabolic stress, structural remodeling, and tissue injury, rather than from measuring aging in a purely statistical sense. At the same time, not all proteins within these modules should be interpreted equally, as some function primarily as biomarkers or readouts of damage, whereas others may represent candidate mediators of age-related decline (Sayed et al., 2021; Oh et al., 2023; Argentieri et al., 2024).

From a biomarker perspective, shared and organ-enriched proteomic signatures address different clinical questions. A multi-process proteomic score may provide a sensitive summary of overall biological aging burden and facilitate population-level risk stratification, whereas organ-enriched markers may offer greater localization. The potential value of an integrative score is illustrated by the proteomic age clock developed using Olink measurements from 45,441 UK Biobank participants. The resulting 204-protein model predicted chronological age with a Pearson correlation of 0.94 and retained similar age-prediction accuracy in independent Chinese (n = 3,977; r = 0.92) and Finnish (n = 1,990; r = 0.94) cohorts. Proteomic age acceleration was also associated with incident diseases across multiple organ systems, multimorbidity, and all-cause mortality (Argentieri et al., 2024). These findings support the generalizability of an integrative proteomic aging signature across geographically and genetically diverse populations.

The same breadth, however, limits diagnostic specificity. IL-6, CRP, GDF15, and complement proteins can be elevated by infection, obesity, renal dysfunction, cardiovascular disease, cancer, or medication exposure, making it difficult to attribute an abnormal shared-module score to one organ or mechanism. Conversely, organ-enriched signatures or injury markers such as NT-proBNP and NfL offer greater localization but may miss coordinated deterioration outside the target system. A potential hierarchical framework would therefore combine these complementary levels of information: a shared multi-process score could first estimate systemic aging burden, followed by organ-enriched protein panels, imaging, or conventional clinical tests to localize the dominant sources of dysfunction. Longitudinal within-person measurements, multivariable adjustment for comorbidities, and models that jointly partition shared and organ-specific variance will be necessary to disentangle the relative contributions of individual systems (Oh et al., 2023; Argentieri et al., 2024).

## 8 Proteomic technologies and associated challenges in aging proteomics

Proteomic profiling in aging research relies on a heterogeneous ecosystem of analytical platforms, each with distinct technical constraints that shape sensitivity, specificity, dynamic range, and ultimately biological interpretability (Hortin and Sviridov, 2010; Ignjatovic et al., 2019; Kirsher et al., 2025). Broadly, current approaches can be divided into untargeted and targeted modalities, representing different stages along the biomarker discovery-to-validation continuum (Ignjatovic et al., 2019; Kirsher et al., 2025). Untargeted mass spectrometry (MS) enables hypothesis-free exploration and provides peptide-level structural resolution, including the detection of post-translational modifications (PTMs) and protein isoforms (Angel et al., 2012; Ignjatovic et al., 2019). However, its effective dynamic range remains substantially narrower than that of the plasma proteome, whose protein abundances span more than ten orders of magnitude, limiting comprehensive detection of low-abundance cytokines, growth factors, and tissue-leakage proteins in unfractionated plasma (Hortin and Sviridov, 2010; Angel et al., 2012). A comparison of major proteomic platforms used for circulating protein profiling, including their analytical principles, coverage, strengths, limitations, and representative references, is summarized in Table 2.

**TABLE 2:** Comparison of major proteomic platforms used for circulating protein profiling.

Platform	Analytical principle	Typical blood proteome coverage	Representative strength in aging studies	Major limitation in aging proteomics	References
Antibody arrays	Solid-phase antibody capture array	up to ∼4,000 targets	customizable profiling; modification-capable; scalable screening	epitope dependence; antibody quality; relative quantification	Huang et al. (2010), Huang and Zhu (2017), Chen et al. (2018)
Olink	Proximity extension assay (PEA)	∼3,000–5,400 targets, depending on panel version	high sensitivity; low sample input; cohort-friendly; standardized service workflows	predefined panels; relative NPX output; binder dependence	Wik et al. (2021), Haslam et al. (2022), Sissala et al. (2025)
SomaScan	Aptamer-based affinity proteomics	∼7,000–11,000 targets, depending on version	broad target coverage; high throughput; aging clock applications; standardized fee-for-service access	aptamer specificity; proteoform sensitivity; limited cross-platform concordance	Pietzner et al. (2021), Haslam et al. (2022), Oh et al. (2023)
Mass spectrometry (MS)	Peptide-based untargeted or semi-targeted proteomics LC–MS/MS with sequence-linked detection	∼400–6,000 proteins*	unbiased discovery; PTM/proteoform analysis; mechanistic insight	limited plasma depth; lower sensitivity for low-abundance proteins; expensive instrumentation and specialized expertise; lower cohort scalability	Hortin and Sviridov (2010), Ignjatovic et al. (2019), Beimers et al. (2025)

MS, coverage varies substantially with depletion, fractionation, enrichment, and acquisition workflow. Emerging multi-nanoparticle protein-corona and Mag-Net enrichment workflows can increase proteome depth but introduce workflow-specific selection and recovery biases (Blume et al., 2020; Ferdosi et al., 2022; Wu et al., 2025).

**FIGURE 2:**
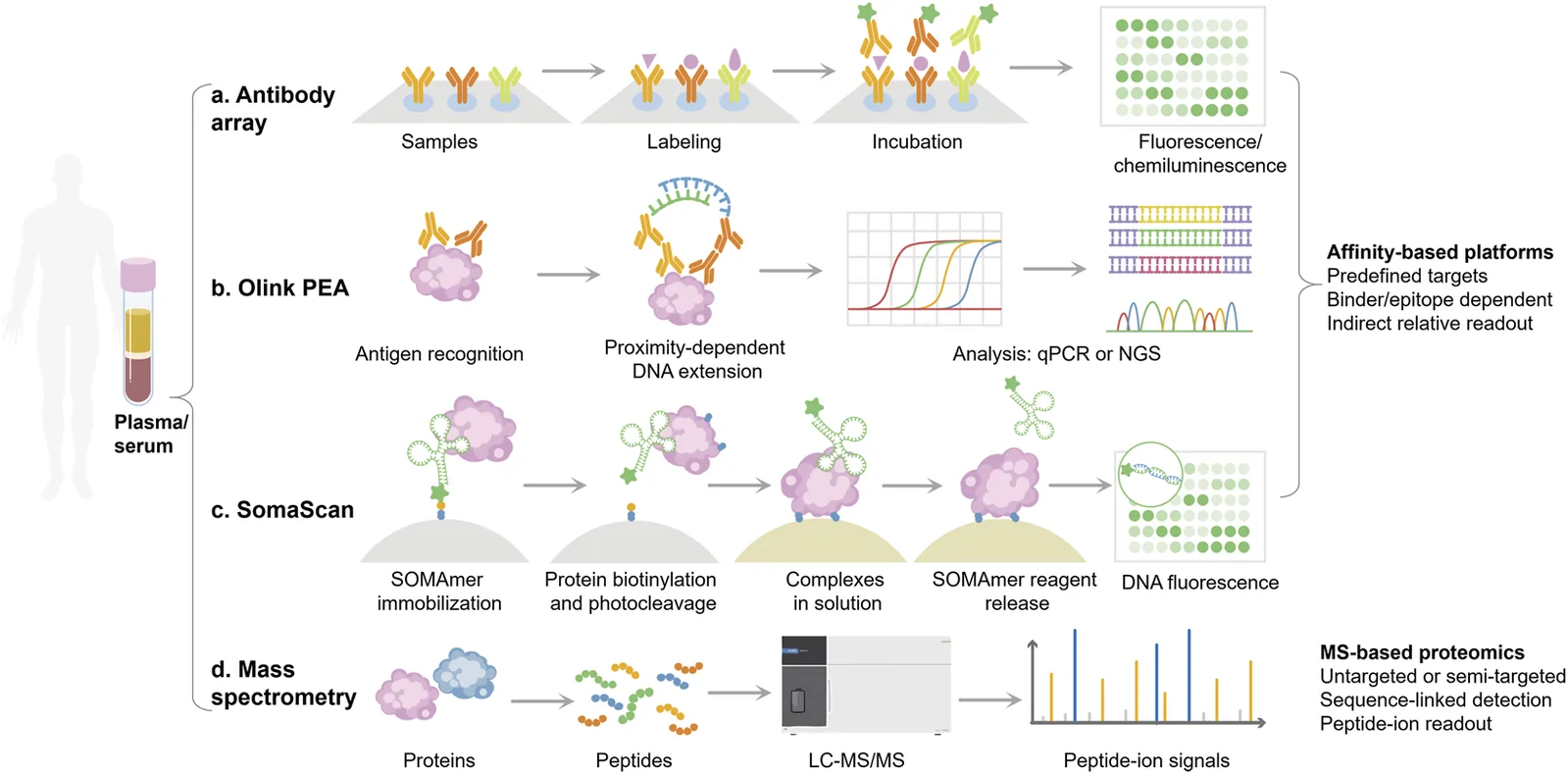
Overview of major proteomic platforms used in aging research. Schematic sample-to-readout workflows of representative platforms for profiling circulating proteins in plasma or serum. **(a)** Antibody arrays use immobilized capture antibodies and direct or secondary detection, with fluorescence or chemiluminescence as the readout. **(b)** Olink proximity extension assays (PEA) use pairs of oligonucleotide-conjugated antibodies; target-dependent proximity enables DNA extension and subsequent quantification by qPCR or next-generation sequencing (NGS). **(c)** SomaScan uses modified single-stranded DNA aptamers (SOMAmer reagents) for protein binding and DNA-based fluorescence quantification. **(d)** Mass spectrometry (MS)-based proteomics analyzes peptides generated by protein digestion and provides sequence-linked identification and peptide-ion abundance measurements. The first three platforms quantify predefined targets through binder-dependent, indirect relative signals, whereas MS supports untargeted or semi-targeted proteomic profiling based on peptide-sequence evidence.

In contrast, affinity-based multiplex platforms—including aptamer-based assays (e.g., SomaScan), proximity extension assays (e.g., Olink), and antibody arrays—offer very high analytical sensitivity and enable simultaneous quantification of large panels of predefined proteins directly from complex biofluids with minimal sample preprocessing (Huang et al., 2010; Chen et al., 2018; Pietzner et al., 2021; Haslam et al., 2022). These technologies are therefore particularly well suited for large aging cohorts and longitudinal studies. Nevertheless, their reliance on predefined binders introduces intrinsic epitope dependence: changes in protein conformation, proteolytic processing, sequence variation, or age-associated PTMs may alter binding affinity, producing apparent abundance shifts that do not necessarily reflect true protein concentration (Santos and Lindner, 2017; Pietzner et al., 2021; Sissala et al., 2025). This issue is especially relevant in aging, where oxidative modifications, glycosylation changes, and broader proteoform remodeling are pervasive (Baldensperger et al., 2020; Johnston and Buckley, 2023). An expanded overview of these major proteomic technologies and their complementary roles across the biomarker discovery-to-validation continuum is shown in Figure 2.

SomaScan uses chemically modified single-stranded DNA aptamers (SOMAmers) to recognize predefined protein targets, with recovered aptamers quantified by DNA microarray fluorescence. Olink proximity extension assays use pairs of oligonucleotide-conjugated antibodies; dual target binding enables DNA extension and subsequent quantification by qPCR or next-generation sequencing, with results reported as normalized protein expression (NPX) values. Antibody arrays immobilize capture antibodies on a solid surface and quantify retained targets through direct labeling or secondary detection. Thus, affinity platforms convert binder recognition into an indirect relative signal, whereas MS measurements are anchored to peptide-ion signals and sequence evidence (Huang et al., 2010; Chen et al., 2018; Pietzner et al., 2021; Haslam et al., 2022). However, MS workflows require specialized instrumentation and substantial expertise in sample preparation, chromatography, instrument operation, data analysis, and quality control. By contrast, Olink and SomaScan are often accessible through standardized centralized or fee-for-service workflows, facilitating their implementation in large cohort studies (Pietzner et al., 2021; Haslam et al., 2022). The approaches should therefore be viewed as complementary: MS is advantageous for unbiased discovery, proteoform characterization, and PTM mapping, whereas affinity platforms enable scalable measurement of predefined, often low-abundance proteins across large cohorts.

Emerging enrichment strategies are helping to narrow the depth gap in MS-based plasma proteomics. Multi-nanoparticle workflows exploit the selective adsorption of plasma proteins onto engineered nanoparticle surfaces to form distinct protein coronas. Combining coronas generated by particles with different physicochemical properties compresses the effective dynamic range and enriches proteins that are poorly detected in unfractionated plasma, while remaining compatible with automated LC–MS/MS workflows (Blume et al., 2020; Ferdosi et al., 2022). A related but conceptually distinct approach, Mag-Net, uses strong-anion-exchange magnetic beads to enrich extracellular vesicles and other membrane-bound particles before digestion, thereby improving the detection of vesicle- and membrane-associated proteins that fall below the detection range of unfractionated plasma LC–MS/MS (Wu et al., 2025). These approaches increase proteome depth and support high-throughput sample processing, although their selective enrichment profiles, recovery biases, and protocol dependence should be considered when comparing results across studies.

Technical variability further complicates aging proteomics. Batch effects arising from reagent lots, instrument drift, sample handling, and processing time can approach or exceed biological effect sizes, particularly in longitudinal studies where age-related changes accumulate gradually (Hassis et al., 2015; Ignjatovic et al., 2019). Affinity assays also introduce platform-specific normalization schemes, such as relative NPX values in proximity extension assays, which limit absolute comparability across studies (Wik et al., 2021; Haslam et al., 2022). In addition, preanalytical variables—including processing delays, sample handling, and broader participant-related factors such as inflammatory status, medication exposure, and comorbidities—can substantially influence circulating protein measurements and therefore require rigorous quality control and statistical adjustment.

A persistent challenge across all platforms is cross-technology concordance. Benchmarking studies often show limited overlap in detectable proteins and only modest correlation for shared targets between MS-, aptamer-, and antibody-based systems (Huang and Zhu, 2017; Pietzner et al., 2021; Sissala et al., 2025). These discrepancies arise from fundamental differences in measurement principles, dynamic range, epitope accessibility, and analytical workflows, complicating meta-analysis and cross-platform integration.

A further conceptual challenge in aging proteomics is the existence of proteoforms—the distinct molecular forms of a protein produced by genetic variation, alternative splicing, proteolytic processing, and combinations of PTMs (Santos and Lindner, 2017). Measured protein signals therefore do not always map straightforwardly onto total biological abundance (Pietzner et al., 2021; Allgoewer et al., 2023). In older individuals, proteins are increasingly subject to oxidation, glycation, truncation, altered glycosylation, and other proteoform-level changes. Affinity reagents may recognize one shared epitope, preferentially bind a particular proteoform, or lose binding when that epitope is modified (Pietzner et al., 2021; Sissala et al., 2025). Bottom-up MS provides sequence-specific peptide evidence and can directly identify many PTMs, but protein digestion may prevent reconstruction of the complete intact proteoform when its defining modifications occur on different peptides (Bludau et al., 2021). Consequently, an apparent age-associated increase or decrease may represent a composite of altered abundance, structural remodeling, or differential assay accessibility. This issue helps explain why platforms may agree at the level of phenotypic prediction while showing only modest concordance for nominally identical protein targets (Pietzner et al., 2021; Sissala et al., 2025). Such divergence does not necessarily invalidate proteomic aging signatures, but it does require careful interpretation of what a given biomarker or protein clock is actually measuring.

Analytical robustness also depends on prespecified statistical design. Covariate adjustment in age–protein association analyses should be guided by the causal question and may include major biological and clinical factors such as sex, ancestry, adiposity, renal function, inflammatory status, medication use, and comorbidities, while avoiding indiscriminate adjustment for variables that may lie on the causal pathway. False-discovery-rate control is also required when testing thousands of proteins simultaneously (Benjamini and Hochberg, 1995; VanderWeele, 2019). Longitudinal studies should use mixed-effects or trajectory models to account for repeated measurements and non-linear age trends, with explicit separation of within- and between-person effects when relevant; time-to-event models are appropriate for incident outcomes (Curran and Bauer, 2011). For proteomic clocks, preprocessing, feature selection, and hyperparameter tuning should be confined to training and resampling loops, followed by calibration and independent validation against functional or clinical outcomes (Kapoor and Narayanan, 2023; Collins et al., 2024). Cross-study replication additionally requires harmonized protein mappings and phenotype definitions, transparent normalization and batch correction, and explicit assessment of platform-related heterogeneity (Čuklina et al., 2021; Eldjarn et al., 2023).

Collectively, age-associated proteomic signatures are inseparable from the technologies and analytical models used to generate them, with important implications for biomarker discovery and the interpretation of proteomic aging clocks and cross-system aging modules. Reproducible aging biomarkers therefore benefit from complementary measurement approaches, transparent quality control, independent validation, harmonized normalization, and explicit consideration of platform-specific biases (Pietzner et al., 2021; Kirsher et al., 2025; Sissala et al., 2025).

## 9 Future directions

Advances in high-throughput proteomics have transformed our understanding of human aging, revealing dynamic, system-level protein signatures that reflect biological age rather than chronological age (Argentieri et al., 2024; Kliuchnikova et al., 2024). Despite rapid progress, several scientific, technical and translational challenges remain before proteomic biomarkers can be fully integrated into clinical practice (Haslam et al., 2022; Sissala et al., 2025). Future research must therefore focus on improving biomarker precision, expanding longitudinal datasets, advancing mechanistic interpretation, and developing clinically actionable tools for risk prediction and intervention monitoring.

First, there is a critical need for standardization of proteomic platforms, protocols, and analytical pipelines (Haslam et al., 2022; Sissala et al., 2025). Differences among mass spectrometry, SomaScan, Olink, and antibody-based assays introduce substantial variability that complicates cross-study comparisons and clinical translation (Pietzner et al., 2021; Haslam et al., 2022). Future work should prioritize harmonized calibration standards, reference protein panels, and technical reproducibility benchmarks across laboratories. This includes establishing population-specific normative ranges and age-related trajectories for key proteins such as GDF15, CXCL9, NfL, IGF-1, and inflammatory cytokines. The adoption of international quality-control consortia, similar to genomics and metabolomics, will be essential to create stable cross-platform interoperability (Sissala et al., 2025).

Second, future directions should focus on conducting deep longitudinal phenotyping to define individual aging trajectories (Kliuchnikova et al., 2024; Tang et al., 2025). Many current studies use cross-sectional sampling, which makes it difficult to distinguish between intra-individual and inter-individual variability. Repeated multi-timepoint measurements across decades will allow the development of personalized proteomic aging curves, identification of early divergence signals before clinical disease onset, and quantification of “resilience” after physiological stressors (Tang et al., 2025). Integrating proteomic profiles with wearables, imaging, digital phenotyping, and electronic health records will accelerate the discovery of protein markers that dynamically track functional capacity, frailty transitions, and organ-specific decline (Oh et al., 2023; Tang et al., 2025).

Third, the mechanistic understanding of how circulating proteins drive or reflect aging remains incomplete (Kliuchnikova et al., 2024). Many highly predictive biomarkers, including GDF15, VCAM-1, CXCL9, matrix metalloproteinases, and complement factors, function as integrative signals of mitochondrial stress, inflammation, or extracellular matrix remodeling, yet their causal roles are still not fully resolved (Conte et al., 2022; Kliuchnikova et al., 2024). Future work should therefore combine functional genomics, perturbation-based approaches, and proteo-transcriptomic integration to map regulatory networks linking circulating proteins to cellular aging pathways (Liu et al., 2022). Emerging technologies, including organoid systems, single-cell proteomics, and spatial proteomics, should further improve mechanistic resolution of tissue-specific contributions to the systemic aging proteome.

Fourth, proteomic data offer substantial potential for developing clinically useful biological age clocks (Argentieri et al., 2024; Wang et al., 2024b). However, existing algorithms remain limited by cohort specificity, risks of overfitting, and incomplete biological interpretability (Wang et al., 2024b). Next-generation clocks should incorporate multi-system protein modules, causal biological pathways, and organ-specific signals rather than relying solely on statistical associations (Oh et al., 2023). Importantly, these models should be validated not only for chronological age prediction, but also for morbidity, treatment responsiveness, and healthspan-related outcomes (Yu et al., 2025). Linking proteomic clocks to intervention studies involving senotherapeutics, rapalogs, metformin-related strategies, caloric-restriction mimetics, exercise interventions, and microbiome-targeted therapies may help determine whether biological age is modifiable and whether proteomic signatures can serve as surrogate markers of geroprotective efficacy (Franceschi et al., 2019; Yu et al., 2025).

Finally, future directions should place greater emphasis on equity, diversity, and global applicability. Although recent large-scale studies have begun to validate proteomic aging models across geographically and genetically diverse populations, substantial parts of the current evidence base remain dominated by European and North American cohorts, limiting the generalizability of existing biomarkers and aging clocks (Argentieri et al., 2024). Large-scale multi-ethnic cohorts, studies in underrepresented settings, and explicit evaluation of cross-population aging trajectories are therefore needed to improve clinical relevance. Future models should also incorporate sex, ancestry, environmental exposures, and socioeconomic context, as these factors may shape age-related proteomic variation and influence the interpretation of biological aging.

In summary, proteomics offers an unprecedented opportunity to quantify systemic aging, uncover mechanistic drivers of decline, and support more precise prevention strategies for age-related diseases. Continued innovation in technology, cohort design, computational modeling, and clinical validation will pave the way toward proteome-informed strategies that enhance healthspan and resilience across the human lifespan.

## 10 Conclusion

Proteomics provides a systems-level framework for understanding aging by capturing coordinated molecular changes across major physiological systems, including the immune, metabolic/endocrine, cardiovascular, musculoskeletal, and nervous systems. Large-scale cohort studies have shown that circulating proteins exhibit quantifiable age-related trajectories and are closely associated with biological age, multimorbidity, functional decline, and mortality risk (Oh et al., 2023; Argentieri et al., 2024). Across these systems, recurring biological processes—including chronic inflammation, metabolic and mitochondrial stress, endocrine remodeling, extracellular matrix remodeling, and neuro-glial injury—support the existence of shared cross-system protein aging modules.

Importantly, proteomic aging signatures should not be interpreted simply as statistical predictors of chronological age. Their biological value lies in integrating multiple layers of systemic aging, distinguishing biomarkers or readouts from candidate mediators, and linking molecular alterations to organ-level vulnerability and functional decline. Looking forward, longitudinal and multi-omics integration will be essential for refining causal interpretation and translating proteomic aging models into clinically meaningful tools. Overall, proteomics is shifting aging research from single-pathway descriptions toward an operational systems-biology framework for measuring and understanding human aging.
